#  Pesquisa social em saúde: por que ler Norbert Elias?

**DOI:** 10.1590/0102-311XPT049023

**Published:** 2023-12-22

**Authors:** Rosana Magalhães

**Affiliations:** 1 Escola Nacional de Saúde Pública Sergio Arouca, Fundação Oswaldo Cruz, Rio de Janeiro, Brasil.

**Keywords:** Ciências Sociais, Agenda de Pesquisa em Saúde, Pesquisa, Social Sciences, Health Research Agenda, Research, Ciencias Sociales, Agenda de Investigación en Salud, Investigación

## Abstract

Este ensaio busca refletir sobre os desafios teórico-metodológicos do campo da pesquisa social em saúde e, especialmente, explorar a matriz conceitual presente na obra de Norbert Elias, sociólogo e historiador cuja abordagem sobre o processo civilizador contribui substancialmente para a compreensão das singularidades que conformam a existência individual e coletiva. Investigando longas cadeias de interdependência no decorrer da história, Elias desafiou interpretações deterministas sobre os processos sociais e construiu análises expressivas sobre uma pluralidade de objetos em perspectiva interdisciplinar, construtivista e relacional. Ao iluminar a dinâmica de interação e constituição mútua dos indivíduos e da sociedade, o autor redefine a natureza das relações recíprocas entre as estruturas sociais, a economia psíquica e o corpo. Nesse sentido, a apropriação dessa postura reflexiva pode originar estudos e processos de investigação. Considerando a complexidade da Saúde Coletiva, propõe-se, assim, estimular possíveis articulações, diálogos e usos do referencial teórico eliasiano na problematização e construção de estratégias e caminhos de pesquisa.

## Abstract (en)

This essay aims to reflect on the theoretical-methodological challenges of social research in health and, especially, to explore the conceptual matrix present in the work of Norbert Elias, a sociologist and historian whose approach to the civilizing process greatly contributes to understanding the singularities that shape individual and collective existence. By investigating long chains of interdependence throughout history, Elias challenged deterministic interpretations of social processes and built a vigorous analysis of a plurality of objects from an interdisciplinary, constructivist, and relational perspective. By illuminating the dynamics of interaction and the mutual constitution of individuals and society, the author redefines the nature of the reciprocal relations among social structures, the psychic economy, and the body. In this sense, the appropriation of this reflexive perspective can fertilize studies and research processes. Considering the complexity of Public Health, we propose to stimulate possible articulations, dialogues, and uses of the Eliasian theoretical framework in the problematization and construction of strategies and research paths.

## Resumen

El ensayo busca reflexionar sobre los desafíos teórico-metodológicos del campo de la investigación social en salud y, sobre todo, explorar la matriz conceptual presente en la obra de Norbert Elias, sociólogo e historiador cuyo enfoque sobre el proceso civilizador aporta una gran contribución a la comprensión de las singularidades que configuran la existencia individual y colectiva. Investigando largas cadenas de interdependencia a lo largo de la historia, Elias desafió interpretaciones deterministas sobre los procesos sociales y construyó un análisis vigoroso sobre una pluralidad de objetos desde una perspectiva interdisciplinaria, constructivista y relacional. Al iluminar la dinámica de interacción y constitución mutua de los individuos y de la sociedad, el autor redefine la naturaleza de las relaciones recíprocas entre las estructuras sociales, la economía psíquica y el cuerpo. En este sentido, la apropiación de esta postura reflexiva puede fertilizar estudios y procesos de investigación. Considerando la complejidad de la Salud Colectiva, se propone incentivar posibles articulaciones, diálogos y usos del marco teórico eliasiano en la problematización y construcción de estrategias y caminos de investigación.

## Introdução

As experiências de saúde e doença são fortemente associadas à dinâmica das relações sociais. Há uma vasta literatura internacional e nacional sobre como o acesso à educação básica de qualidade, moradia, saneamento, trabalho e participação política têm peso decisivo nas condições de saúde ^1^^,^^2^^,^^3^^,^^4^. Além da privação absoluta de bens e serviços, a privação relativa também impacta os níveis de saúde da população. Países com maior igualdade no que se refere à distribuição de renda, por exemplo, apresentam maior esperança de vida ao nascer. Relações de gênero também alteram as práticas de saúde e repercutem em riscos diferenciados de doença. Nos Estados Unidos, os homens morrem, em média, sete anos antes das mulheres e têm taxas de mortalidade mais altas em todas as 15 principais causas registradas ^5^. Ações, mecanismos e estratégias utilizados para a afirmação da feminilidade e da masculinidade tendem a contribuir para a manutenção de maiores ou menores níveis de autocuidado. Como analisa Tilly ^6^, crenças de gênero apoiadas em regras culturais com maior grau de institucionalização estimulam a construção de estereótipos que se traduzem em barreiras para o acesso aos recursos relevantes. Com isso, podemos dizer que disposições singulares para lidar com o corpo, acesso a recursos e distintos riscos à saúde emergem de maneira complexa e dinâmica.

Desvantagens sociais associadas à raça e etnia também repercutem nas taxas de morbidade e mortalidade. No estudo de Farmer & Farraro ^7^, adultos negros apresentam doenças mais graves e piores autoavaliações de saúde em comparação com adultos brancos. No Brasil, vários autores sublinham os diferenciais entre brancos e negros em relação à mortalidade infantil, mortalidade de jovens, prevalência de doenças crônicas, acesso a serviços de saúde e itinerários terapêuticos ^8^^,^^9^. Coesão social e vínculos comunitários também influenciam os padrões de saúde e doença.

Esse debate orientou o movimento da Medicina Social, no século XIX e nas décadas seguintes, e contribuiu para a crítica à concepção estritamente biomédica dos fenômenos ligados à saúde e à doença. Houve aproximação com as bases epistemológicas da Sociologia, da Antropologia, da História, da Economia e outras disciplinas. O diálogo entre as Ciências Sociais e a saúde ocorreu em diferentes países com diversos níveis de institucionalização, impactos e influências teóricas. Nesse percurso, a concepção materialista da História, elaborada por Karl Marx e Friedrich Engels, e o foco no papel das forças produtivas e da estrutura de classes na conformação dos padrões de organização social tornaram-se importantes ferramentas heurísticas. A obra clássica de Engels sobre as condições de vida da classe trabalhadora na Inglaterra durante a Revolução Industrial estimulou a reflexão sobre as relações entre a dinâmica capitalista, a pobreza e a saúde. Mas, especialmente após a Segunda Guerra Mundial, a abordagem sociológica das iniquidades em saúde estabeleceu fortes relações com o pensamento de Émile Durkheim e a perspectiva estrutural-funcionalista. Assim, a centralidade das instituições, das normas e dos valores para a manutenção da estabilidade, integração e ordem social, presente em Durkheim, ganha ressonância na elaboração da doença como processo disfuncional.

Já o estudo de Talcott Parsons, no início dos anos 1950, enfatizou o peso das macroestruturas externas ao indivíduo na conformação das condições de saúde e a imagem do adoecimento como desvio. Nos anos 1960, o interacionismo simbólico e as contribuições de Erving Goffman e Howard Becker, nos Estados Unidos, por exemplo, tensionaram a visão dos indivíduos passivos e submetidos inexoravelmente às forças coercitivas do sistema social ^10^. Em grande parte, a abordagem weberiana e a chamada Sociologia compreensiva sobre o sentido da ação dos agentes sociais e o conflito de interesses provocaram importantes deslocamentos no debate. Sob a influência dessa tradição teórica, surgiram muitos estudos voltados à problematização das questões de gênero, raça, identidade sexual e suas interfaces com a saúde.

Na verdade, sem a pretensão de esgotar a análise dessa verdadeira “batalha de paradigmas” e de seus múltiplos desdobramentos, podemos dizer que as fronteiras e conexões entre as ações dos indivíduos e as condições estruturais sob as quais se dão tais ações foram objeto de reflexão e disputa intelectual permanente entre os cientistas sociais. Essa complexidade e, muitas vezes, ambivalência também marcaram as estratégias interpretativas acerca da realidade social da saúde.

Como analisou Canesqui ^11^, no Brasil, a construção do campo da Saúde Coletiva, na década de 1970 e nos anos seguintes, revelou a expansão da articulação entre as Ciências Sociais e a saúde com diferentes ênfases dadas à reflexão teórica, à relevância das bases empíricas, ao uso de indicadores qualitativos e quantitativos e aos múltiplos significados das práticas. Para Maria Andréa Loyola & Maurício Barreto ^12^ (p. 24), “*as ciências sociais constituem o cerne mesmo, o coração e a própria razão de ser da Saúde Coletiva*”. De fato, pesquisadores preocupados com a construção de um olhar mais abrangente buscaram escapar das dualidades entre indivíduo e sociedade, natural e social, subjetividade e objetividade, quantitativo e qualitativo e, nessa trajetória, formularam conceitos e metodologias que apoiam a investigação científica sobre as múltiplas dimensões das experiências e práticas que conformam o campo da Saúde Coletiva.

Evidentemente, essa articulação entre as Ciências Sociais e a saúde não é isenta de contradições e controvérsias. As tensões entre a visão da Medicina como Ciência Social e o enfoque clínico e individual, capaz de isolar e tratar agentes patogênicos, não foram completamente dissipadas e, de certa forma, ainda limitam a discussão contemporânea sobre a saúde. Apesar das evidências de que a saúde não é afetada apenas por questões biológicas, mas também pela posição social e por diferentes perfis de interação institucional, política e simbólica, o desenho de programas explicativos e estratégias de pesquisa interdisciplinares visando dar conta dessa problemática é desafiante. Em grande parte, a pluralidade de tendências e correntes analíticas que caracterizam as Ciências Sociais gera, para o campo da Saúde Coletiva, incertezas, dificuldades e potencialidades. Assim, podemos assumir que dilemas e confrontos paradigmáticos ligados à miríade de enfoques que habitam as Ciências Sociais também estão presentes nos estudos e nas investigações sobre saúde e doença.

Certamente foge ao escopo deste artigo revisar profundamente os estudos que abordaram a constituição do campo da Saúde Coletiva e os múltiplos desafios da pesquisa social em saúde ^13^^,^^14^^,^^15^. Mas é importante ressaltar algumas tendências e questões relevantes. Como apontam Minayo et al. ^16^, o problema do determinismo que assombrou as Ciências Sociais nos anos 1970, a ilusão da cientificidade apoiada preferencialmente em dados estatísticos e a visão fragmentada e tecnicista marcaram essa trajetória. Ao mesmo tempo, o subjetivismo e a ênfase nos processos microssociológicos representaram, muitas vezes, um distanciamento reflexivo de questões mais amplas e estruturais em torno das experiências e práticas de saúde.

Não há dúvida de que a Saúde Coletiva e a pesquisa social em saúde impõem, permanentemente, investimentos em teorização. Esse esforço, porém, não deve alimentar expectativas em torno de um modelo analítico definitivo. Sabemos que o discurso científico, ainda que possa enriquecer a análise do mundo social, não é o espaço de verdades absolutas, mas de contribuições sempre parciais. Para Canesqui ^17^ (p. 62), “*a recriação de novas sínteses sobre um objeto híbrido como a saúde coletiva, não dilui as tensões. Recriam-se novas e certamente não se abarcam todas as possibilidades abertas às idéias e práticas acumuladas, assim como às que ainda advirão*”.

Nessa direção, o diálogo com as Ciências Sociais seguirá controverso, ainda que inescapável. Neste artigo, o objetivo é examinar possibilidades e estratégias exploratórias, fazendo uso da reflexão sobre o universo conceitual e metodológico presente na obra de Norbert Elias. Considerando o consenso inexistente, a provisoriedade e, também, as limitações do processo de produção de conhecimento no campo da Saúde Coletiva, a perspectiva é favorecer e estimular a aproximação, com um enfoque interdisciplinar e relacional, para apoiar a problematização e a fertilização da pesquisa na área.

## Interdependência, configuração e equilíbrio de tensões: a Sociologia Reflexiva de Norbert Elias

Para Garrigou & Lacroix, a obra de Norbert Elias “*tem um objeto se não exclusivo, pelo menos central: a história; uma problemática: a sociologia; e uma orientação: uma teoria da política*” ^18^ (p. xxix). Assumindo tal envergadura, como elaborar uma síntese do pensamento do autor? Além disso, como criar um diálogo entre tais referências e disposições conceituais e a pesquisa social em saúde? Para avançar nessa tarefa, é importante, inicialmente, explorar alguns dos principais eixos teóricos e ferramentas metodológicas propostas por Elias.

Como ponto de partida, um dos fundamentos da obra é a profunda conexão entre processos sociais e biológicos, interpretada com base no estudo de longos percursos históricos na França, Alemanha, Inglaterra e Gana. Mediante a noção de interdependência, Elias revela a relação indissociável entre as transformações da estrutura econômica e política na sociedade de Corte no Ocidente europeu e a emergência de uma nova economia psíquica. Sob a análise de Elias ^19^ (p. 155), “*o padrão de controle das inclinações, do que deve e não deve ser controlado, regulado e transformado, certamente não é o mesmo* (...) *conforme os diferentes tipos de interdependência, a sociedade burguesa aplica restrições mais fortes a certos impulsos, ao passo que certas restrições, que eram aristocráticas, são transformadas para se adaptarem à nova situação*”.

Evitando a polarização entre a conformação de regularidades sociais e motivações individuais e valorizando o ecletismo metodológico, a investigação social de Elias explora, de maneira articulada, a psicogênese e a sociogênese. Estruturas sociais são instituídas a partir de indivíduos interdependentes. Ou seja, recusando a ideia de uma sociedade sem indivíduos e vice-versa, o autor integra o desenvolvimento dos corpos e organismos humanos ao desenvolvimento do espaço social e emergência de novos arranjos de poder.

Para isso, há o uso do que ele chamou de “armas da história”. Elias analisa, com base em documentos e registros históricos abarcando vários séculos, a centralização do poder em torno do rei, o surgimento da burguesia, do monopólio fiscal e da violência legítima exercida pelo Estado e, ao mesmo tempo, a organização de uma nova gestão dos afetos. Nesse percurso, marcado por padrões complexos de dependência e interação entre os indivíduos e grupos, cada vez mais é necessário lidar com as pulsões, encontrar formas de autocontrole e transformar qualitativamente as relações sociais. Se antes, nas sociedades feudais, as “explosões de fúria”, o prazer com a tortura e a violência eram comuns e esperadas, com o surgimento de uma nova aristocracia, no âmbito das Cortes absolutistas, começam a ser construídos padrões distintos de vergonha, embaraço e pacificação. As pressões por reconhecimento e *status* emergem em um contexto de competição transformado, e suavizar o comportamento, internalizando novos códigos de conduta, torna-se crucial para a conquista de maior mobilidade social. Como analisa Heinich ^20^, funções entendidas como naturais são remodeladas, e uma nova sensibilidade, envolvendo a progressiva aversão aos fluidos do corpo, à falta de pudor e às pulsões, é articulada ao contexto histórico e social de transição política e econômica. Recusando as armadilhas do racionalismo e do determinismo, tais mudanças são entendidas como processos não planejados, mas, ainda assim, plenos de significados e intencionalidades: “*o irrevogável entrelaçamento dos atos, necessidades, idéias e impulsos de muitas pessoas dá origem a estruturas e transformações estruturais numa ordem e direção específicas que não são simplesmente animais, naturais ou espirituais, nem tampouco racionais ou irracionais, mas sociais*” ^21^ (p. 39). O processo civilizador é compreendido como uma rede de fluxos interdependentes, na qual, em vez de elementos isolados, emergem relações. Esse fluxo é sustentado por laços de reciprocidade, padrões de dependência e entrelaçamento das pessoas de maneira cooperativa, mas também por meio de impulsos coercitivos, desequilíbrios de poder e resistências. Surge, assim, o conceito de configuração para interpretar a dinâmica entre indivíduos e sociedade e designar as mudanças na estrutura das emoções, nas formas de convivência e nas diferentes práticas sociais: “*o que temos em mente com o conceito de configuração pode ser convenientemente explicado com referência às danças de salão* (...)*. Pensemos na mazurca, no minueto, na polonaise, no tango ou no rock’n’roll. A imagem de configurações móveis de pessoas interdependentes na pista de dança talvez torne mais fácil imaginar Estados, cidades, famílias e, também, sistemas capitalistas, comunistas e feudais como configurações* (...)*. As mesmas configurações podem certamente ser dançadas por diferentes pessoas, mas, sem uma pluralidade de indivíduos reciprocamente orientados e dependentes, não há dança. Tal como todas as demais configurações sociais, a da dança é relativamente independente dos indivíduos específicos que a formam aqui e agora, mas não de indivíduos como tais*” ^19^ (p. 250).

Ao analisar a configuração da sociedade de Corte, a monopolização da violência pelo Estado e novas redes interdependentes, Elias percebe um controle mais efetivo das emoções, ainda que difícil e balizado pelas diferentes tradições históricas de cada contexto. Esse comportamento, chamado pelo autor de civilizado em contraponto aos modos de interação do regime feudal, não é harmônico ou definitivo. Tampouco o autor sucumbe ao evolucionismo. Movimentos civilizatórios e descivilizatórios irão se alternar, comportamentos brutalizados podem prevalecer, especialmente em situações em que não existam esferas centralizadas e legitimadas de poder. Há claramente, nas sociedades europeias analisadas e posteriormente em outros contextos, um “equilíbrio de tensões”. Isso porque, ao longo da história, os fenômenos da urbanização, da comercialização e da centralização do poder vão atingir a maioria dos países ocidentais. Com isso, emergem novas relações de cooperação que exigem autocontrole, além de habilidades não violentas, com o objetivo de reestruturar os arranjos de poder frente a uma distinta e densa ordem de dependências recíprocas.

Temos, assim, um claro esforço de superação das oposições clássicas entre indivíduo e sociedade, natureza e cultura, corpo e mente, na medida em que, para Elias, o indivíduo é constituído na cadeia de relações sociais em que vive: “*não existe um grau zero de vinculação social do indivíduo, um começo ou ruptura nítida em que ele ingresse na sociedade como que vindo de fora, como um ser não afetado pela rede, e então comece a se vincular a outros seres humanos*” ^21^ (p. 31). De acordo com o autor, o modo como as características de uma criança serão aos poucos cristalizadas não depende de uma natureza ou constituição inicial, como uma planta que tem um caminho de maturação único a partir de uma semente. Existe, na criança, ao contrário, o que Elias chama de “*uma profusão de individualidades possíveis*” ^21^ (p. 28). Será, portanto, na dinâmica dos intercâmbios e das relações com outras pessoas que a criança se torna adulta e é, consequentemente, conformada à estrutura de indivíduo. Nessa direção, sociedades e indivíduos não são “ontologicamente” distintos.

Apesar de seu estudo sobre o processo civilizador ter sido concluído em 1939, essa abordagem implicou certo isolamento e difusão lenta de sua obra na medida em que não acompanhava as linhas interpretativas hegemônicas nas Ciências Sociais até o fim dos anos 1970.

## A utilização do referencial analítico eliasiano na pesquisa em saúde

Como vimos, Elias chama a atenção para a dinâmica de entrelaçamento entre a existência individual e a social. Para o autor, não há um “eu” destituído do “nós”. Nessa perspectiva, os múltiplos sentidos sobre o corpo, o psiquismo e a identidade individual são construídos e reconstruídos nas redes de interdependência que conformam as diferentes configurações sociais. O processo de interiorização dos constrangimentos e de desenvolvimento do autocontrole corrobora para a formação de corpos “civilizados” em contextos sociais concretos.

Analisando a contribuição dessa abordagem no campo da saúde, Freund ^22^ chamou a atenção para o avanço e complexificação da divisão do trabalho e das cadeias de interdependência ao longo da história e, concomitantemente, sobre como Elias articula esse movimento à necessidade de racionalizar o tempo e, consequentemente, os processos sociais, biológicos e físicos. Assim, nas sociedades modernas, emerge um habitus temporal, no qual as fronteiras entre impulsos, funções fisiológicas, instintos e demandas sociais são fluidas.

Para Elias ^23^ (p. 79), “*o estudo do tempo é de uma realidade inserida na natureza, e não de uma natureza e uma realidade humana separadas*”. A cisão entre o tempo, como dimensão física medida por fórmulas matemáticas, e a “instituição social do tempo”, que regula os eventos e a ação humana, gera uma enorme dificuldade para a compreensão das conexões entre natureza e sociedade. Assim, como não deve existir oposição conceitual entre o tempo físico e o tempo vivido, é preciso reconhecer a interdependência entre o biológico e o social. Diferentemente da ideia de um corpo biológico fixo, Elias afirma a existência de experiências corporais sempre abertas a mudanças e reposicionamentos. Equilíbrios e desequilíbrios se alternam, conflitos entre os ritmos do corpo e as exigências sociais são permanentes. Nessas circunstâncias, o que podemos chamar de “comportamentos saudáveis” não são objeto de escolhas pessoais ou de uma ordenação das práticas alheia aos processos de socialização. Na verdade, a busca de um balanço positivo nas condições de saúde envolve a construção de arranjos para lidar com riscos, contextos sociais e recompensas subjetivas. Elias utiliza o conceito de “economia psíquica” exatamente para explorar a construção dessa trajetória humana até a vida adulta, na qual será possível a internalização de mecanismos de autocontrole no âmbito do processo civilizatório. Nas últimas décadas, muitos estudos sobre obesidade, doenças mentais, mortalidade por acidentes de trânsito, prevenção ao câncer, violência, envelhecimento e processos de estigmatização associados à saúde têm avançado com base no referencial analítico proposto por Elias.

As pesquisas de Stuij ^24^ e Porter ^25^ sobre os múltiplos desdobramentos do processo civilizador na saúde associaram o ganho de peso e a obesidade às formas cada vez mais diferenciadas e complexas de autocontrole, desenvolvidas em sociedades marcadas pelo aumento da oferta calórica em vez da escassez. Paradoxalmente, esse aumento da densidade calórica tende a ocorrer, sobretudo, em alimentos ultraprocessados e com menor valor aquisitivo, ameaçando a saúde das populações mais pobres. A valorização da magreza revelaria, assim, expectativas em torno do corpo singulares, históricas e, principalmente, uma combinação peculiar de racionalização, fluxos mercadológicos e gestão das pulsões em contextos obesogênicos. Nesse cenário, as mulheres, que proporcionalmente foram ainda mais afetadas pelo processo de monitoramento e controle das emoções ao longo da história, tendem a ser mais impactadas. Vergonha, embaraço e culpa em torno do corpo obeso expressariam, assim, as múltiplas faces do processo de interiorização de constrangimentos e de uma autoimagem depreciada ^26^.

No que se refere à saúde mental, o estresse crônico, associado às expectativas e limitações existentes nas sociedades contemporâneas, impacta a produção hormonal e gera a chamada “fadiga emocional”, sobretudo nas situações em que há múltiplas demandas e pouca autonomia decisória. Além disso, evidências sobre o desenvolvimento do cérebro humano demonstram a importância dos primeiros anos de vida, nos quais os circuitos neurológicos recebem estimulação e são fortalecidos. Rupturas familiares, violência e insegurança tendem a comprometer esse processo. Ainda que existam fatores hereditários, diferentes pesquisas apoiadas no referencial eliasiano revelam a forte influência do padrão de interação social vivido na infância no surgimento de doenças mentais, dependência de drogas e inúmeras formas de sofrimento psíquico ^27^.

O aumento dos acidentes de trânsito e o impacto nas taxas de mortalidade também foram objeto de pesquisas orientadas pela perspectiva teórica de Norbert Elias. Com base nesse referencial, compreende-se que, se, por um lado, os carros favorecem a mobilidade, por outro, envolvem vigilância e autocontrole consideráveis, principalmente nos contextos urbanos. Crescem, nesse caso, exigências em torno do uso de tecnologias, respeito a regras e sinais de trânsito e, também, adaptações frente à presença de ciclistas, pedestres e condições estruturais das estradas. Tais situações podem desencadear “surtos descivilizatórios”. O motorista, portanto, não é um indivíduo isolado, autônomo e descontextualizado. Assim como hábitos alimentares, comportamento sexual e formas de expressão das emoções, a performance no trânsito envolve potencialidades biológicas, orientações culturais e transformações na organização social de maneira interdependente ^28^.

As iniquidades em saúde associadas à raça também podem ser interpretadas a partir da perspectiva eliasiana sobre os processos de estigmatização e contra-estigmatização. Para Elias, o próprio uso do termo raça e o foco na cor da pele revela a dificuldade para superar a ênfase em questões periféricas para compreender relações de poder e configurações. Seu interesse intelectual concentra-se, então, na dinâmica em que se dá a construção de relações “racializadas” e nos momentos de ruptura. Como analisa Fletcher ^29^, quando o balanço de poder é tensionado e favorece grupos em desvantagem social, sentimentos de inferioridade são transformados, e novos cenários e possibilidades de integração e democratização do acesso a recursos e bens relevantes podem surgir.

Para Pinnell ^30^, o diagnóstico e tratamento do câncer com maiores chances de cura implicam estabelecimento de uma rotina de exames preventivos, os quais, por sua vez, são movidos por uma disposição para o autocuidado. Dificilmente os indivíduos adotam tais práticas apenas com base em mensagens e indicações formais dos profissionais e instituições de saúde. Isso porque as decisões sobre a saúde não derivam, em grande parte, de uma maior racionalidade ou do reconhecimento dos avanços científicos. Referências simbólicas e uma certa reflexividade, associadas aos padrões de engajamento nos circuitos e cadeias de interação social, teriam, portanto, maior peso na detecção precoce da doença. Ou seja, “*a capacidade para interpretar as manifestações do corpo corretamente e reconhecer sintomas e potenciais indicações de um câncer em estágio inicial, requer não somente conhecimento, mas também uma disposição psíquica peculiar*” ^30^ (p. 13).

O apelo para o aumento da atividade física com o objetivo de reduzir a prevalência de diferentes morbidades - um verdadeiro mantra na agenda das políticas públicas de promoção da saúde - tem sido reinterpretado à luz do referencial analítico proposto por Elias. O imperativo da vida saudável tende a generalizar a visão das pessoas como indivíduos isolados, motivados e plenamente capazes de fazer escolhas. De fato, a crescente previsibilidade da vida social contemporânea e a longevidade vis-à-vis o contexto pré-moderno apresentam novas possibilidades para a prevenção de diferentes riscos e agravos à saúde. Mas, para Elias, o que está permanentemente em jogo na conformação dos corpos e das coletividades é a radical interdependência entre funções fisiológicas e práticas sociais. A reflexão do autor sobre o processo de esportificação, desenvolvido em parceria com Eric Dunning ^31^, fornece pistas importantes para a compreensão das novas formas de prazer, excitação e alívio associadas aos exercícios físicos. Assim, à medida que pulsões e instintos são controlados e os contextos sociais tornam-se menos violentos, o incremento de atividades físicas e a prática de esportes revelariam mais uma face do “impulso civilizador”.

Nesse sentido, para além das prescrições com base no discurso científico ou nas distintas experiências de adoecimento, a adesão ao exercício físico envolve lidar com representações simbólicas do corpo, oportunidades para liberar emoções espontâneas, processos de estigmatização, expectativas associadas à posição social e chances de gratificação emocional. Para Gibson & Malcolm ^32^ (p. 11), no contexto contemporâneo, “*ser ativo fisicamente é ser saudável, ser saudável é ser normal ainda que ironicamente* (...) *ser ativo não é normal (estatisticamente). Projetando a inatividade como resposta desviante no que tange aos comportamentos desenvolvidos no cenário contemporâneo (crescente uso da internet, automação, etc.) a política de promoção da atividade física representa uma racionalização, na perspectiva eliasiana exigência de autocontrole sobre comportamentos guiados por bases biológicas e/ou aprendidos socialmente*”.

No Brasil, alguns estudos e pesquisas sobre atividade física, violência, envelhecimento e morte têm utilizado a abordagem de Norbert Elias e contribuído para a compreensão dos processos relacionais que envolvem tais temas. No entanto, podemos dizer que ainda é tímido o diálogo com a obra no campo da Saúde Coletiva. Um grande desafio para a generalização de um olhar mais abrangente e menos instrumental é a compreensão dos múltiplos e complexos mecanismos que operam de maneira imbricada e não linear na saúde dos indivíduos e grupos sociais. Como salientaram Faria et al. ^33^ (p. 3), “*nos anos de 1980, quando Elias publicou sua obra sobre o envelhecer e a morte, os debates estavam centrados nos aspectos fisiológicos e patológicos...*”. Sem dúvida esse é, ainda, um importante obstáculo não totalmente superado. Além disso, cabe chamar a atenção para os limites da chamada “determinação social da saúde da doença”, uma tendência analítica que aglutina diversos pesquisadores e estudiosos na área. O grande mérito dessa abordagem é, sem dúvida, introduzir, no debate sobre a saúde, as dimensões da classe social, do gênero, da raça, do território e dos perfis institucionais, entre outras. Como apresentado no início deste ensaio, evidências das complexas associações entre a saúde e as condições de vida são obtidas por meio de estudos e pesquisas com esse enfoque. Com isso, os limites do modelo biomédico são tensionados e torna-se possível investigar as conexões entre condições estruturais, privações simbólicas e processos de adoecimento. No entanto, seguindo os passos de Elias, é importante incorporar na agenda de pesquisa a ênfase em correlações e interdependências em vez de determinações. Como afirma Blondel ^34^ (p. 51), “*as noções de necessidade causal, de determinismo, oriundas das experiências físico-químicas, podem até entravar a pesquisa e levar a designar como causa e efeito fenômenos interdependentes*”.

No estudo etnográfico realizado por Elias em uma pequena cidade inglesa, no fim da década de 1950, é possível perceber uma crítica robusta à transformação das propriedades estruturais em fatores ou variáveis isoladas e, ao mesmo tempo, à imagem das pessoas como seres submetidos às “determinações sociais e econômicas”. Para Elias ^35^, os diferenciais de poder entre estabelecidos e *outsiders* evidenciam um padrão, ao mesmo tempo, estável e mutável, no qual as influências da ordem social são interiorizadas, enfrentadas e ressignificadas pelos agentes. O grande problema ligado ao determinismo é, portanto, o obscurecimento de mudanças e novos arranjos de poder que surgem no processo de entrelaçamento entre agência e estrutura. Ainda que existam estudos sofisticados sobre os efeitos recíprocos entre regularidades sociais e a saúde dos indivíduos, uma narrativa que evite a perspectiva da “determinação” pode avançar na compreensão das diferentes configurações locais e representar importantes ganhos cognitivos no âmbito das atividades de pesquisa.

## Pontes, afinidades e convergências intelectuais

Não é de se admirar que uma obra preocupada em articular diferentes saberes e avançar na interdisciplinaridade apresente uma ampla interlocução com diferentes matrizes e correntes analíticas. Há, nos textos de Elias, um “ecumenismo vocabular e conceitual” e incorporação de autores como Marx, Durkheim, Weber e Freud. As limitações disciplinares para a compreensão das pressões civilizatórias e descivilizatórias e, também, das resistências que repercutem no corpo, nas emoções e nas estruturas sociais, ganhando novas traduções e contornos em cada contexto singular, foram enfrentadas pelo autor com recursos teóricos oriundos da Antropologia, da Psicanálise, da História e da Sociologia. A originalidade, porém, é percebida na busca de um *framework* capaz de apreender correlações, interações, contingências e acasos por meio de conceitos relacionais.

Nessa perspectiva, a Sociologia Configuracional de Elias estabeleceu pontes e convergências intelectuais com vários autores. Para Freund ^22^, uma importante característica dos estudos de Erving Goffman é o foco na “corporificação” dos acontecimentos relacionados à interação social na vida cotidiana. Essa perspectiva microssociológica é relevante para compreender a dramatização das experiências relacionadas ao processo civilizador analisado por Elias.

Podemos dizer que o conceito de *habitus*, desenvolvido por Pierre Bourdieu, o qual busca apoiar a reflexão sobre gostos, disposições e inclinações operando como uma linguagem distintiva e princípio de classificação, tem clara proximidade com as noções utilizadas por Elias para dar conta das relações complexas entre o mundo subjetivo e objetivo. Na verdade, o termo é utilizado por Elias e descrito pelo autor como “*a composição social dos indivíduos*” ^21^ (p. 150). Para Shilling ^36^, enquanto uma teoria do corpo como capital físico é encontrada em Bourdieu, Elias estaria preocupado com a construção de um esquema conceitual capaz de dar conta do corpo civilizado. Portanto, ambos os autores contribuem consideravelmente para o estudo do corpo e da construção da identidade na sociedade contemporânea. As lutas por legitimidade e distinção associadas a estratégias de autocontrole, em situações marcadas por desequilíbrios de poder, podem ser descritas como pontos de interesse em comum. Nas palavras de Bourdieu ^37^ (p. 48), “*a corte tal como Elias a descreve é um belíssimo exemplo do que chamo um campo em que, como um campo gravitacional, os diferentes agentes são arrastados por forças insuperáveis, inevitáveis num movimento perpétuo necessário para manter hierarquias, distâncias e afastamentos*”.

A visão de uma modernidade não planejada, o entrelaçamento entre influências globalizantes e disposições individuais e a noção da “dualidade da estrutura”, presentes em Giddens ^38^^,^^39^, estabelecem um diálogo com a crítica de Elias ao funcionalismo e aos sentidos dados à ação humana e às coerções sociais no pensamento sociológico ortodoxo. Os sensos de orgulho e vergonha, que acompanham o projeto reflexivo do eu e do corpo, e os diferentes fluxos de dependência que integram a experiência cotidiana nas sociedades contemporâneas foram questões amplamente discutidas por ambos os autores.

Há, então, um horizonte de interlocução consistente, e os pesquisadores que se debruçaram sobre os processos de individualização, transformação do tecido social, estigmatização, contra-estigmatização e exclusão encontraram afinidades intelectuais e produziram um rico diálogo com a obra de Elias.

## Conclusão

Elias construiu estratégias interpretativas, apoiadas em uma perspectiva relacional, que ultrapassam antagonismos entre o corpo, os indivíduos e a dinâmica social. Processos interdependentes, influências recíprocas e produção de padrões de interação conflitivos e cooperativos se tornaram os principais eixos de sua análise sobre rupturas, continuidades e transições históricas em diferentes sociedades. Reconhecendo o necessário engajamento entre pesquisadores e seus objetos de pesquisa e, ao mesmo tempo, a importância do distanciamento crítico, Elias contribuiu para a articulação entre as dimensões macro e microssociológicas e para a valorização dos estudos empíricos orientados por esquemas conceituais robustos. Ao teorizar sobre relações de poder, disposições corporais e ordenamentos sociais, o autor evitou a especulação abstrata, a naturalização dos processos sociais, o reducionismo, o determinismo e a supremacia do método.

Considerando as profundas diferenças entre o que tem significação estatística e o que tem significação sociológica, Elias desenvolveu uma perspectiva interdisciplinar sobre múltiplos objetos de pesquisa e enfrentou o desafio de relativizar o poder explicativo de estudos baseados apenas em métodos quantitativos ou modelos experimentais. Nesse sentido, o autor pode ser considerado uma importante referência para interpretar as experiências e a dinâmica institucional no campo da Saúde Coletiva. A pesquisa na área entendida como prática social envolve a interpretação dos contextos históricos em que corpos são conformados e traduzem emoções, revelam identidades e expressam diferentes capacidades de ação. Funções fisiológicas e aprendizados sociais podem ser interpretados, portanto, como dimensões articuladas na medida em que processos de socialização e exigências corporais se entrelaçam e produzem valores e expectativas historicamente singulares. Isso implica dizer que não basta acumular dados empíricos ou buscar associações do tipo causa e efeito. Sem um quadro de análise consistente e conceitos “relacionais”, dificilmente será possível compreender disposições, constrangimentos estruturais, resistências e oportunidades para deliberações reflexivas associadas à saúde nos diferentes contextos sociais.
